# Correction: Individual control of singlet lifetime and triplet yield in halogen-substituted coumarin derivatives

**DOI:** 10.1039/d0ra90101f

**Published:** 2020-10-01

**Authors:** Katrin E. Oberhofer, Mikayel Musheghyan, Sebastian Wegscheider, Martin Wörle, Eleonora D. Iglev, Rositca D. Nikolova, Reinhard Kienberger, Petko St. Petkov, Hristo Iglev

**Affiliations:** Physics Department E11, Technical University of Munich James-Franck-Str. 85748 Garching Germany hristo.iglev@tum.de; Faculty of Chemistry and Pharmacy, University of Sofia J. Bouchier 1 1164 Sofia Bulgaria ppetkov@chem.uni-sofia.bg; W. L. Gore & Associates GmbH Hermann-Oberth-Str. 22 85640 Putzbrunn Germany

## Abstract

Correction for ‘Individual control of singlet lifetime and triplet yield in halogen-substituted coumarin derivatives’ by Katrin E. Oberhofer *et al.*, *RSC Adv.*, 2020, **10**, 27096–27102, DOI: 10.1039/D0RA05737A.

The authors regret that the name of one of the authors (Petko St. Petkov) was shown incorrectly in the original article. The corrected author list is as shown above.

The Royal Society of Chemistry apologises for these errors and any consequent inconvenience to authors and readers.

## Supplementary Material

